# Local recurrence and locoregional metastases as precursors of death by cutaneous squamous cell carcinoma

**DOI:** 10.1111/ddg.15902

**Published:** 2025-10-25

**Authors:** Helmut Breuninger, Thomas Kurt Eigentler, Rolf Ulrich, Klaus Dietz

**Affiliations:** ^1^ Department of Dermatology University Hospital Tübingen Tübingen Germany; ^2^ Department of Dermatology Venereology and Allergology Charité – Universitätsmedizin Berlin Berlin Germany; ^3^ Department of Psychology University of Tübingen Tübingen Germany; ^4^ Department of Medical Biometry (Emeritus) University of Tübingen Tübingen Germany

**Keywords:** Cutaneous squamous cell carcinoma, metastasis, prognosis, recurrence

## Abstract

**Background:**

In cutaneous squamous cell carcinoma (cSCC), there is a paucity of data regarding local recurrence (LR) and regional lymph node metastasis (LM) as precursors to tumor death.

**Patients and Methods:**

We estimated parameters of a multistate model with a proportion of patients without risk of progression using maximum likelihood. For seven risk factors, the relative risks of progression to LR, LM, and LR&LM were compared with the relative risks of cSCC death among patients with progression.

**Results:**

Of the 1,400 patients, 124 (8.9%) were diagnosed with either LR (68), LM (42), or LR&LM (14). cSCC‐specific death occurred in 33 patients (2.4%), 10 by LR, 14 by LM, and 9 by LR&LM, among these 10 distant metastases. Conversely, 493 patients (35.2%) died from other causes. The risk of death from cSCC was 14.7% for LR, 33.3% for LM, and 64.3% for LR&LM. The median time to disease‐related death was 1.3 years.

**Conclusions:**

The risk of progression is limited to approximately 25% of patients. LM has been found to be associated with a higher risk of disease‐specific death in comparison to LR. The median interval from progression to disease‐specific death was found to be 1.3 years.

## INTRODUCTION

Cutaneous squamous cell carcinoma (cSCC) is one of the malignant skin tumors whose incidence is increasing significantly due to an aging population and increased recreational sun exposure.^1^ Unfortunately, there is a lack of disease‐specific registries to evaluate the outcomes of cSCC, as several tumor entities besides cSCC, such as basal cell carcinoma or Merkel cell carcinoma, are grouped in registries under ICD‐10 C44.^2^

In addition, due to the advanced age of patients with cSCC, compliance with follow‐up is very limited. At this age, many patients have serious comorbidities that can lead to immobility or even death. In the latter case, it is sometimes not easy to distinguish whether patients have died from the tumor or with the tumor.

The current *American Joint Committee on Cancer* (AJCC) classification system for non‐melanoma skin cancers poses a challenge to clinicians and researchers alike.^3^ While it provides a framework for classifying and treating these cancers, it often lacks specificity, especially when it comes to the subtypes and variations of cSCC that occur in clinical practice. Factors such as tumor size, tumor thickness (in mm), depth of invasion, degree of differentiation, desmoplasia, and perineural infiltration are known to influence prognosis. Still, the existing AJCC classification system does not adequately capture these factors.

We are therefore interested in the value of prognostic factors, especially the occurrence of local recurrence and locoregional metastasis, as indicators of death in patients with cSCC. In addition, we would like to consider known risk factors for metastasis more thoroughly in terms of their validity in relation to tumor‐specific death.

## PATIENTS AND METHODS

The present study is based on the same prospectively investigated 1,400 patients that we described in detail in our previous publication.^4^ Only tumors treated for the first time in our department were evaluated. Tumors that were already advanced at the time of initial diagnosis were excluded. Many patients later developed another, prognostically less favorable cSCC at another site, with 11 additional, clearly distinguishable cSCC‐specific deaths.

The progression of each tumor was tracked prospectively using a follow‐up system based on the following data sources: High‐risk patients were followed up at the center in accordance with the applicable guidelines. If a face‐to‐face visit was not possible, a prepaid, regular follow‐up questionnaire was sent for completion by either the general practitioner or the patient, inquiring about the course and outcome of the disease.

In addition, the database of the interdisciplinary *Comprehensive Cancer Center* (CCC) of the University of Tübingen, which contained follow‐up data from other medical institutions and the death register of the state of Baden‐Württemberg, was synchronized with our data.

The initial diagnosis of cSCC was made clinically and/or histologically. Clinical examinations and an ultrasound of the regional lymph nodes were performed. The tumors (median diameter 16 mm [2.00–180 mm]) were treated surgically under local tumescent anesthesia and subsequently underwent complete three‐dimensional margins‐control (3D histology) with embedding in paraffin, hematoxylin‐eosin staining and accelerated fixation at 60°Celsius, so that the result was available within 24 hours.^5^, ^6^, ^7^ This method has proven to be highly sensitive in detecting tumor extension in epithelial tumors without increasing the effort.^8^, ^9^ For tumor‐positive margins, R0 resection was performed whenever possible, even in bone with histological control. If the tumor progressed, surgical treatments were continued until R0 was reached or a surgically incurable R1 condition developed. In the latter case, or if surgical incurability was present from the outset, radiotherapy or chemotherapy was carried out individually. Effective immunotherapies were not yet used at that time.

Age, gender, immunosuppression, comorbidities, and clinical tumor diameter were documented. The histological diagnosis was carried out by specialized dermato‐histopathologists. It included the histological tumor thickness, infiltration depth, differentiation grade I–III (III = poor), desmoplasia, and perineural invasion with measurement of nerve thickness which includes desmoplasia.^10^ During the course, satellite and in‐transit metastases, as well as local recurrence, lymph node metastasis, and distant metastasis, were registered.

We used the *JMP 17.2 statistical package* (SAS Institute Inc., Cary, NC) to analyze the nonparametric distributions of five competing risks using the lifetime distribution menu. First, we examined local recurrence, lymph node metastases, and local recurrences together with lymph node metastases, accounting for deaths from other causes and censoring, but excluding subsequent events. In the online supplement, we describe the likelihood function of the multi‐state model along with parameter estimates.

For seven dichotomous risk factors, we calculated the relative risks for the occurrence of local recurrence, lymph node metastases, and local recurrences together with lymph node metastases, as well as for cSCC‐specific death only for patients with progression, to compare the effect of the risk factors before and after progression. The product of these two relative risks is the relative risk of cSCC‐specific death at diagnosis. The confidence intervals for the relative risks were calculated using the *DescTools program* of the *R statistical package* employing the “score” method.^11^ Significance was tested using the Pearson test. To correct the significance level for the number of tests performed per progression type, we used the Bonferroni‐Holm method.^12^

Our evaluation was approved by the independent ethics committee of the University Hospital of Tübingen (reference number: 568/2016BO1) and conducted in accordance with the *STROBE guidelines for observational studies*. Patients were informed about the purpose of the study and signed a standardized consent form to participate in data collection.

## RESULTS

**FIGURE 1:**
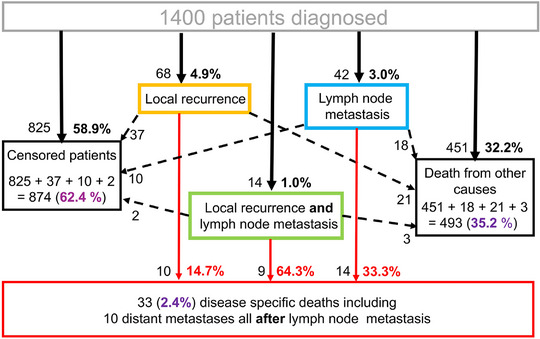
States and transitions of 1,400 cSCC patients in our study. The percentages of the five initial events are shown in black digits. The percentages of the three final events are shown in purple digits. The percentages of disease‐specific deaths per progression type are shown in red digits.

The events of local recurrence, lymph node metastasis, and their combination in cSCC, which occurred in 124 of the 1,400 patients (8.8%) after diagnosis, are presented in full in Figure 1. The majority of patients, 874 (62.4%), were censored (left black box). Local recurrence alone was observed in 68 cases (orange box, 54.8% of 124) and directly resulted in disease‐specific death due to local incurability in 10 patients (red box). The number of censored patients was 37, while 21 of the patients with local recurrence died of other diseases (right black box).

Lymph node metastases alone occurred in 42 patients (blue box, 33.9% of 124), of which 14 led to two types of disease‐specific death (4 by death due to incurable lymph node metastasis and 6 by distant metastasis, red box), ten were censored, while 18 of the patients with local recurrence died of other diseases.

Local recurrence together with lymph node metastasis (green box) occurred in 14 cases (11.3% of 124), of which nine resulted in three types of disease‐specific death (1 by local incurability, 4 by incurable lymph node metastasis, and 4 by distant metastasis, red box), two were censored, and three patients died of other diseases. The median time from diagnosis of lymph node metastasis to the development of ten distant metastases was 0.22 years.

**FIGURE 2:**
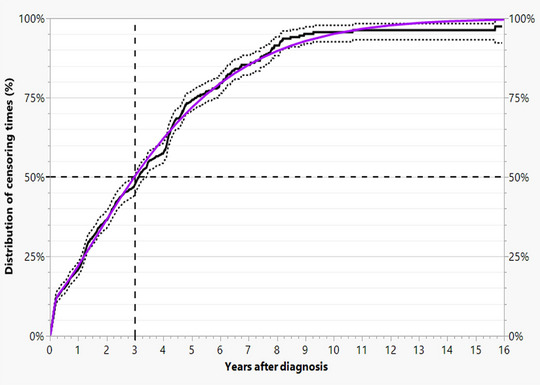
Observed (black continuous step function), 95% confidence intervals (dashed step functions), and fitted mixture of Weibull distributions (purple curve) of censoring times with a median of 3 years.

**FIGURE 3:**
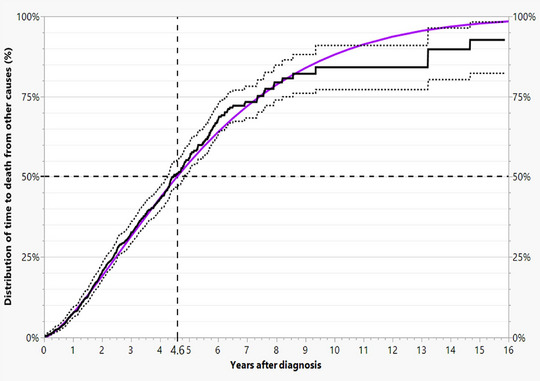
Observed (black continuous step function), 95% confidence intervals (dashed step functions), and fitted Weibull distribution (purple curve) of times of death from other causes with a median of 4.6 years.

To describe the incidence of progression, we calculated the time from diagnosis to the three types of progression using a multistate model. We consider death from other causes (32.2%) and censoring (58.9%). In the present data, the latter ranged from two days to 15.9 years (median 3 years). About 11% of patients are censored within approximately 11 weeks (Figure 2). The median time to death by other causes is 4.6 years. (Figure 3)

**FIGURE 4:**
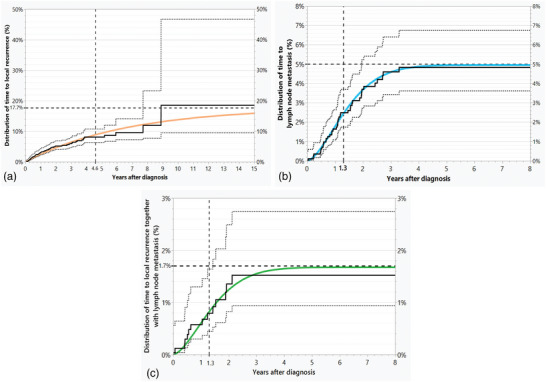
(a–c) Observed distributions of progression times (black continuous step functions) together with 95% confidence limits (black dashed step functions) as estimated considering competing risks, together with the estimated distributions (continuous curves in color) with numerical estimates of the medians and the estimated cumulative percentage.

The non‐parametric estimates of the distributions for local recurrence, lymph node metastasis, and local recurrence together with lymph node metastasis reach a maximum well below 100%, suggesting that only a small proportion of patients were at risk of progression. (Figure 4a–c). According to the parameter estimates of our model, 75.7% of all patients had no risk of progression. Among the remaining patients at risk (n = 340), only 36.5% experienced progression (124 cases), owing to censoring and deaths from other causes. Table 1 shows the estimated distribution of progression types at diagnosis and their fitted final distribution together with the observed final distribution, which is in very good agreement with each other, providing evidence for the model.

**TABLE 1:** Estimated distribution of progression types at diagnosis and their fitted final distribution together with the observed final distribution.

Initial states			Final states				
	*Estimated initial distribution*		*Local recurrence*	*Local recurrence and lymph node metastasis*	*Lymph node metastasis*	*Death from other causes*	*Censored patients*
Without risk neither for local recurrence nor for lymph node metastasis	1,059.9 (75.7%)					379.9	680.0
At risk for local recurrence	340.1 (24.3%)	247.4 (17.7%)	70.3			59.8	117.3
At risk for local recurrence *and* lymph node metastasis		23.4 (1.7%)		14.7		2.4	6.3
At risk for lymph node metastasis		69.3 (4.9%)			43.6	7.0	18.7
Totals estimated	1,400.0 (100.0%)		70.3 5.0%)	14.7 (1.1%)	43.6 (3.1%)	449.1 (32.1%)	822.3 (58.7%)
Totals observed	1,400 (100,0%)		68 4.9%)	14 (1.0%)	42 (3.0%)	451 (32.2%)	825 (58.9%)

The clearly different cumulative distributions of local recurrence, lymph node metastasis, and local recurrence together with lymph node metastasis are shown in Figure 4a–c. Local recurrence (Figure 4a, orange) plateaued at 9 years in 17.7% of all patients. The risk of lymph node metastasis (Figure 4b, blue) was limited to 5.0%. For these patients, the hazard rate was high, with lymph node metastasis occurring within 3.3 years. There were more cases of local recurrence, along with lymph node metastasis, than expected if local recurrence and lymph node metastasis were independent risks. Local recurrences together with lymph node metastases (Figure 4c, green) were limited to 1.7% of patients within 2 years.

**FIGURE 5:**
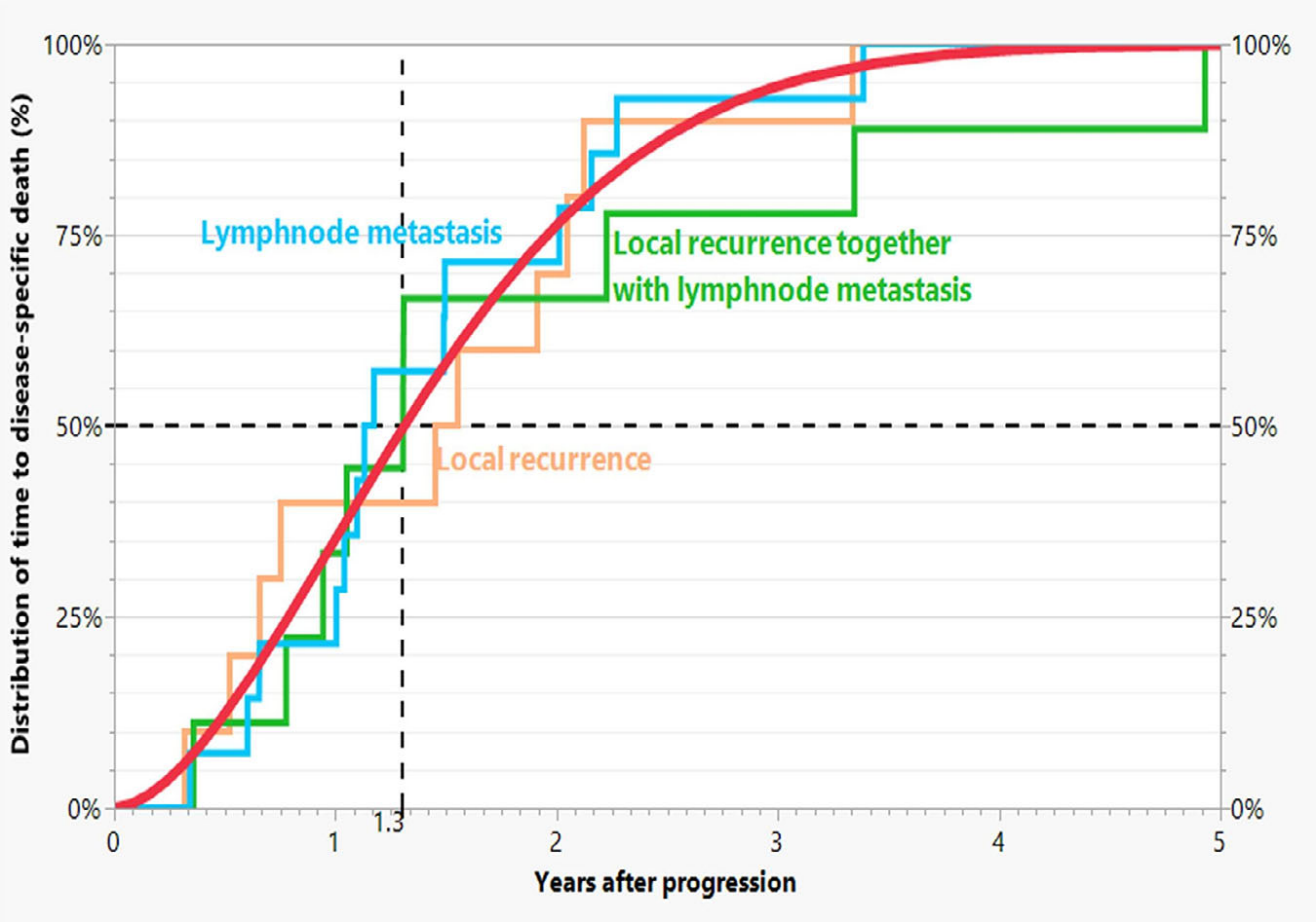
Observed distributions of time to death from onset of progression for 33 patients who died from cSCC for the three types of progression. The fitted Weibull distribution for the types of progression combined has a median of 1.3 years (red curve).

The risk of cSCC‐specific death was 14.7% for local recurrence and 33.3% for lymph node metastasis. When both occurred, the risk of cSCC‐specific death increased to 64.3%. All progressions together had a risk of disease specific death of 26.6%. The time to cSCC‐specific death for all progressions is shown in Figure 5. The median time was 1.3 years.

We estimated the relative risks for seven risk factors discussed in the literature for the different stages of progression: immunosuppression, tumor diameter ≥ 20 mm, tumor thickness ≥ 6 mm, poor differentiation, desmoplasia without and with perineural invasion, perineural invasion and cranial bone invasion before and after the appearance of the different types of progression.^4^, ^13^, ^14^, ^15^, ^16^, ^17^, ^18^

Table 2 shows risk factors for three stages and seven risk factors. Four cells are empty because no disease‐specific deaths occurred for these combinations of progressions and risk factors. Poor differentiation was never significant, because in our pathological classification desmoplasia was a separate risk factor.^10^, ^13^

**TABLE 2:** Risk factors for three stages and seven risk factors. Four cells are empty because no disease‐specific deaths occurred for these combinations of progressions and risk factors.

Numerator/Denominator	Immunosuppression	Diameter ≥ 20 mm	Thickness ≥ 6 mm	Differentiation poor	Desmoplasia. without PNI	PNI & desmoplasia	Bone invasion
68 LR/1,400	2.1	1.4	2.5	1.6	3.5	3.2	3.0
	(1.2–3.6)	(0.9–2.2)	(1.5–3.9)	(0.9–2.8)	(2.0–5.8)	(0.9–9.2)	(1.0–7.6)
10 DSD/68 LR	1.0	0.7	1.5	1.2	2.4	8.3	9.3
	(0.2–3.4)	(0.2–2.0)	(0.5–4.5)	(0.3–4.0)	(0.8–6.7)	(2.5–15.9)	(3.4–18.8)
10 DSD/1,400	2.0	0.9	3.7	1.8	8.1	26.7	28.1
	(0.5–8.2)	(0.3–3.0)	(1.2–12.0)	(0.4–7.6)	(2.5–26.3)	(6.6–93.2)	(8.1–89.8)
42 LNM/1,400	1.9	4.4	4.5	2.3	2.4	2.6	1.6
	(0.9–3.9)	(2.2–8.9)	(2.5–8.1)	(1.2–4.5)	(1.1–5.2)	(0.5–11.8)	(0.3–7.9)
14 DSD/42 LNM	1.2	1.9	0.8	1.3	0.4		3.2
	(0.4–2.7)	(0.6–7.0)	(0.4–1.9)	(0.5–2.9)	(0.1–1.6)		(0.6–5.1)
14 DSD/1,400	2.2	8.3	3.7	3.0	0.9		5.1
	(0.7–7.1)	(2.1–33.2)	(1.4–10.1)	(1.0–8.7)	(0.2–5.5)		(0.9–26.5)
14 LR&LNM/1,400	8.0	2.5	3.7	0.6	6.8	17.8	5.1
	(2.9–21.4)	(0.9–7.1)	(1.4–10.1)	(0.1–3.3)	(2.4–18.8)	(4.6–58.2)	(0.9–26.5)
9 DSD/14 LR&LNM	2.0	0.7	0.8	1.6	0.9	1.7	
	(0.9–5.6)	(0.3–1.7)	(0.3–1.8)	(0.3–2.8)	(0.3–2.0)	(0.6–3.1)	
9 DSD/1,400	15.9	1.7	3.0	0.9	6.1	30.5	
	(4.4–57.6)	(0.5–5.9)	(0.9–10.2)	(0.2–5.6)	(1.7–21.8)	(7.4–109.3)	

*Abbr*.: LR, local recurrence; DSD, disease‐specific death; LNM, lymph node metastasis; PNI, perineural invasion

Before the occurrence of 68 local recurrences, the risk factors of tumor thickness ≥ 6 mm and desmoplasia without perineural invasion were significant; after local recurrence, perineural invasion and bone infiltration were significant. In the overall collective desmoplasia, perineural invasion and cranial bone invasion were significant for the ten disease‐specific deaths.

Before the occurrence of 42 lymph node metastases, tumor diameter, and thickness were significant, after lymph node metastasis, none. In the overall collective, only tumor diameter is significant for the 14 disease‐specific deaths.

Before these 14 disease‐specific deaths local recurrence together with lymph node metastasis, immunosuppression, and desmoplasia without and with perineural invasion were significant. After these events, no factor remained significant. In the overall collective, immunosuppression, desmoplasia, and perineural invasion were significant risk factors for the occurrence of the nine disease‐specific deaths.

## DISCUSSION

In our 2022 publication, which utilized the dataset from this study, the focus was on the risk factors leading to cSCC‐specific death.^4^ Our focus subsequently shifted to progression and the period thereafter (Figure 1). To this end, we incorporated censoring (Figure 2) and deaths from other causes (Figure 3) into the analyses using the multi‐state model described above. This was considered necessary because willingness to undergo follow‐up care declines with age, while age‐related deaths from other causes are approximately 15 times more frequent than disease‐specific deaths.

According to our calculations, approximately three‐quarters of all patients were not at risk of progression. Of the remaining quarter (approximately 340 patients), only 36.5% (124 cases) developed progression, i.e., local recurrence, lymph node metastasis, or both. An overview of progression is shown in Figure 1. Distant metastases occurred only after lymph node metastasis.

The agreement of the estimates with the actual figures supports the model assumptions (Table 1). Figure 4a–c shows that all three types of progression reached a plateau. These had an increasing risk of tumor death in the above order. The median time to cSCC‐specific death after progression was 1.3 years for all (Figure 5). The publication by Wang et al. shows comparable results.^19^

This low risk of progression is achieved by our routine use of the highly sensitive method of complete three‐dimensional margin control (3D histology) with paraffin embedding (see above). 3D histology shows very good results.^9^, ^19^, ^20^ The paraffin technique is essential for extensively infiltrating desmoplastic tumors.^21^ Histologically undetected tumor remnants mean a worse prognosis.^22^ Therefore, all histopathological procedures with diagnostic gaps are obsolete for high‐risk cSCCs.

Risk factors in the classifications usually refer to initial progression.^4^, ^11^, ^12^, ^13^, ^15^, ^16^, ^17^, ^18^ However, once progression has occurred, these factors are often no longer consistently relevant for tumor‐specific death. In Table 2, we show the varying significance of the risk factors according to the three progressions mentioned in various combinations. The risk factor of poor differentiation is never significant because desmoplasia was separated as an independent highly malignant entity.^23^ The other factors appear irregularly. Desmoplasia and the associated perineural infiltration are most frequently significant.

Assessing the prognosis of patients with cSCC remains a challenge, even though the vast majority of patients are at no risk. In contrast to many other tumors, overall survival does not appear to be a valid endpoint for assessing prognosis. Furthermore, the relevance of prognostic factors changes significantly after the first progression. However, the type of progression could be a valid approach for an adequate prognostic classification.

## Conclusions

The risk of progression is limited to about one‐quarter of all patients. Lymph node metastasis is associated with a higher risk of disease‐related death than local recurrence. The median time from progression to disease‐specific death is 1.3 years. Desmoplasia and the associated perineural infiltration are important high‐risk factors .

## CONFLICT OF INTEREST STAEMENT

T.K.E. has received consulting fees from Anaveon, BMS, CureVac, MSD, Novartis, Pierre Fabre, Regeneron, Sanofi and Sun Pharma. All other authors declare no conflict of interest.

## Supporting information



Supplementary information
