# Supplementary material for: Local recurrence and locoregional metastases as precursors of death by cutaneous squamous cell carcinoma
Source: J Dtsch Dermatol Ges. 2025 Oct 25;24(3):319–25. doi: 10.1111/ddg.15902 (PMC12968937; doi:10.1111/ddg.15902)
Supplement: Supplementary file 1 — Supplementary information [file DDG-24-319-s001.docx]

Supporting Information

For the description of the incidence of progression, taking into account censoring and death due to other causes as competing risks, we present a parametric multi-state model for which we estimate the parameters using maximum likelihood.

All time variables can well be described by distributions proposed by W. Weibull. The density is denoted by w(t, sh, sc) with shape parameter sh and location parameter sc, and the cumulative distribution function is denoted by W(t, sh, sc).

The distribution of censoring times (C) is assumed to be a mixture of two Weibull distributions. Therefore, one needs five parameters for their specification: pc is the proportion of very short censoring times (a few weeks) with the Weibull parameters shc1 and scc1. Most censoring times (1-pc) have the Weibull parameters shc2 and scc2.

The distribution of the times of death (DOC) due to other causes has a Weibull distribution with the parameters shd and scd. All individuals are at risk of being censored or dying from other causes.

We need to estimate the at-risk fractions for local recurrence, lymph node metastasis, and local recurrence together with lymph node metastasis, pL, pm, and pLm, respectively.

The time until local recurrence (LR) has an exponential distribution with the parameter scL. The times until the onset of lymph node metastasis (LM) and until local recurrence together with lymph node metastasis (LR˄LNM) are described by the same Weibull distribution with the parameters shm and scm.

We estimate these 5 + 2 + 3 + 3 = 13 parameters according to the method of maximum likelihood

The censoring code (Z) is 1 and the event code is 0.

With these 13 parameters, the probability of each of the five events is given by the following formulas:

Z_DOC = 0 & Z_C = 1 & Z_LR = 1 & Z_LNM = 1 & Z_ LR˄LNM = 1:

w(t,shd,scd)[1–{pcW(t,shc1,scc1)+(1-pc)W(t,shc2,scc2)}][1- pL W(t,1,scL)]

[1–pmW(t,shm,scm)](1–pLmW(t,shm,scm)),

Z_DOC = 1 & Z_C = 0 & Z_LR = 1 & Z_LNM = 1 & Z_ LR˄LNM = 1:

[1-W(t,shd,scd)][pcw(t,shc1,scc1)+(1-pc)w(t,shc2,scc2)][1- pL W(t,1,scL)]

[1-pmW(t,shm,scm)][1-pLmW(t,shm,scm)],

Z_DOC = 1 & Z_C = 1 & Z_LR = 0 & Z_LNM = 1 & Z_ LR˄LNM = 1:

[1-W(t,shd,scd)][1–{pcW(t,shc1,scc1)+(1-pc)W(t,shc2,scc2)}]pLw(t,1,scL)

[1-pmW(t,shm,scm)][1-pLmW(t,shm,scm)],

Z_DOC = 1 & Z_C = 1 & Z_LR = 1 & Z_LNM = 0 & Z_ LR˄LNM = 1:

[1-W(t,shd,scd)][1–{pcW(t,shc1,scc1)+(1-pc)W(t,shc2,scc2)}][1- pL W(t,1, scL)]

pmw(t,shm,scm)[1-pLmW(t,shm,scm)],

Z_DOC = 1 & Z_C = 1 & Z_LR = 1 & Z_LNM = 1 & Z_ LR˄LNM = 0:

[1-W(t,shd,scd)][1–{pcW(t,shc1,scc1)+(1-pc)W(t,shc2,scc2)}][1- pL W(t,1, scL)]

[1-pmW(t,shm,scm)]pLmw(t,shm,scm).

The loss function is – log (likelihood).

Minimizing the loss function yields the following estimates together with their
95% confidence limits:

| **Parameter** | **Estimate** | **95% LL** | **95% UL** |
| --- | --- | --- | --- |
| p_c_ | 10.7% | 9.6% | 11.7% |
| shc1 | 1.83 | 1.69 | 1.97 |
| scc1 (years) | 0.135 | 0.125 | 0.145 |
| shc2 | 1.33 | 1.28 | 1.38 |
| scc2 (years) | 4.48 | 4.35 | 4.62 |
| shd | 1.43 | 1.38 | 1.48 |
| scd (years) | 5.91 | 5.71 | 6.11 |
| p_m_ | 4.95% | 4.16% | 5.74% |
| p_Lm_ | 1.67% | 1.21% | 2.13% |
| p_L_ | 17.7% | 11.3% | 24.1% |
| scL (years) | 6.66 | 3.59 | 9.72 |
| shm | 1.61 | 1.43 | 1.79 |
| scm (years) | 1.65 | 1.43 | 1.87 |

We calculate the expected number of 11 transitions (Table 1) from the four estimated patient groups at the time of diagnosis to the five final states by numerically integrating the product of the respective density function with the probabilities that no censoring and no death from other causes occurred.
